# Protection of Polyphenols against Glyco-Oxidative Stress: Involvement of Glyoxalase Pathway

**DOI:** 10.3390/antiox9101006

**Published:** 2020-10-16

**Authors:** Laura Cianfruglia, Camilla Morresi, Tiziana Bacchetti, Tatiana Armeni, Gianna Ferretti

**Affiliations:** 1 Department of Clinical Sciences, Section of Biochemistry, Biology and Physics, Polytechnic University of Marche, Via Brecce Bianche, 60131 Ancona, Italy; l.cianfruglia@univpm.it (L.C.); g.ferretti@univpm.it (G.F.); 2 Department of Life and Environmental Sciences, Polytechnic University of Marche, Via Brecce Bianche, 60131 Ancona, Italy; c.morresi@univpm.it

**Keywords:** methylglyoxal, glyoxalase system, AGEs (advanced glycation end-products), glutathione, hyperglycemia

## Abstract

Chronic high glucose (HG) exposure increases methylglyoxal (MGO)-derived advanced glycation end-products (AGEs) and is involved in the onset of pathological conditions, such as diabetes, atherosclerosis and chronic-degenerative diseases. Under physiologic conditions the harmful effects of MGO are contrasted by glyoxalase system that is implicated in the detoxification of Reactive Carbonyl Species (RCS) and maintain the homeostasis of the redox environment of the cell. Polyphenols are the most abundant antioxidants in the diet and present various health benefits. Aims of the study were to investigate the effects of HG-chronic exposure on glyco-oxidation and glyoxalase system in intestinal cells, using CaCo-2 cells. Moreover, we studied the effect of apple polyphenols on glyco-oxidative stress. Our data demonstrated that HG-treatment triggers glyco-oxidation stress with a significant increase in intracellular Reactive Oxygen Species (ROS), lipid peroxidation, AGEs, and increase of Glyoxalase I (GlxI) activity. On the contrary, Glyoxalase II (GlxII) activity was lower in HG-treated cells. We demonstrate that apple polyphenols exert a protective effect against oxidative stress and dicarbonyl stress. The increase of total antioxidant capacity and glutathione (GSH) levels in HG-treated cells in the presence of apple polyphenols was associated with a decrease of GlxI activity.

## 1. Introduction

Glyoxalase system is an important enzymatic system implicated in the detoxification of reactive carbonyl species (RCS) such as glyoxal (GO), methylglyoxal (MGO), and 3-deoxyglucosone (3-DG). In general, α-oxoaldehydes are products of glycolytic metabolism and can be formed during lipid peroxidation, or glycation. The effects of MGO, the most reactive metabolite, have been widely studied [1,2]. Accumulation of MGO to toxic level inhibits cell growth and induces cell death [3,4]. In fact, an abnormal accumulation of RCS triggers dicarbonyl stress, resulting in the formation of advanced glycation end-products (AGEs) and DNA modification. MGO can react also with other biomolecules such as nucleotides and basic phospholipids, thus yielding AGEs [5,6]. The increase in RCS contribute to cell and tissue dysfunction and is involved in ageing and in the molecular mechanisms of various chronic disease such as dyslipidemia, obesity, and vascular complications of diabetes [7,8]. 

**Figure 1:**
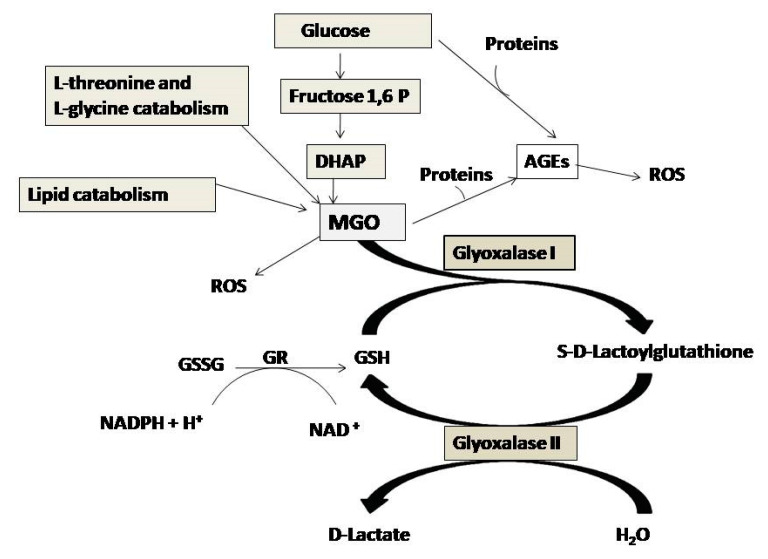
Methylglyoxal (MGO) formation from intermediates of glucose, protein and fat metabolism, and its degradation by the Glyoxalase System. Glyoxalase I (GlxI) converts hemithioacetal formed from glutathione (GSH) and methylglyoxal (MGO) into S-D-Lactoylglutathione (SLG) which is hydrolyzed by Glyoxalase II (GlxII) to D-lactic acid and GSH. Abbreviations: AGEs, advanced glycation end products; DHAP, dihydroxacetone phosphate; ROS, reactive oxygen species; GSH, reduced glutathione; GR, glutathione reductase.

Several studies have shown that the harmful effects of MGO are contrasted by glyoxalase system [9,10]. Glyoxalase system comprises two consecutive enzymes: glyoxalase I (GlxI) and glyoxalase II (GlxII). D-Lactate is a final product and glutathione (GSH) is used as cofactor [11]. Glyoxalase I (EC 4.4.1.5), a lactoylglutathione lyase, catalyzes the isomerization of the hemithioacetal formed non-enzymatically from MGO and reduced GSH to form S-D-lactoylglutathione. Glyoxalase II (EC 3.1.2.6), hydroxyacylglutathione hydrolase, catalyzes the hydrolysis of S-D-lactoylglutathione to D-lactic acid and restores the GSH molecule spent in the first reaction [12] (Figure 1).

Metabolic dysfunction involved in an increase of MGO, and consequently AGEs formation is associated with an increased Reactive Oxygen Species (ROS) formation [13]. Dicarbonyl stress can be increased by oxidative stress. In fact, oxidative stress can lead to a depletion of GSH thus compromising the pathway of glyoxalases that detoxify from MGO by using GSH [14]. Hyperglycemia, associated with diabetes, increased MGO and ROS formation. For instance, incubation of erythrocytes with high concentrations of glucose increases the formation of MGO metabolized to D-lactic acid by the glyoxalase pathway. This increase was proportional to glucose concentrations ranging from 5 to 100 mM and it has been proposed that the glyoxalase system could be implicated in the progress of chronic clinical complications associated with diabetes mellitus [15,16].

Several studies have shown that glyoxalase system has a physio-pathological role in other chronic diseases. Indeed, it was shown that GlxI reduced dicarbonyl and oxidative stress and prevented age-related endothelial dysfunction, a major contributor to cardiovascular disease [17]. Dicarbonyl stress is a mediator that contributes to vascular complications and obesity diabetes-related and is involved in cerebrovascular diseases and neurological disorders [18,19]. Therefore, a growing interest is devoted to molecules able to decrease dicarbonyl stress targeting the glyoxalase system. Our recent study has shown that high glucose concentration triggers an increase of levels of intracellular ROS, lipid peroxidation, and formation of fluorescent AGEs and GA-modified proteins in intestinal cells [20].

Aims of the study were to investigate the effects of high glucose chronic exposure on glyco-oxidation and glyoxalase system in intestinal cells, using CaCo-2 cells. Moreover, we studied the effect of apple polyphenols on glyco-oxidative stress. The CaCo-2 cell line is an important tool in assessing various gastrointestinal functions and is widely used to investigate gastrointestinal oxidant metabolism on a cellular level [21]. In CaCo-2 cells, antioxidant enzymes include glutathione peroxidase, catalase, and paraoxonase-2 (PON2) [22]. Glyoxalase activities have not been studied until today in this cell model. The interest of the study is supported by the physiological relevance of GlxI and GlxII as protective enzymes against glyco-oxidative stress. In fact, intestinal cells are exposed to several diet pro-oxidant factors [23]. The effect of polyphenols on glyco-oxidative stress and glyoxalase has not been studied until today in intestinal cells. A growing interest is deserved to the study of the mechanisms involved in the protective effects exerted by diet polyphenols against glyco-oxidative stress [24,25]. In fact, epidemiological studies and randomized clinical trials have shown a strong association between consumption of polyphenols and reduced risk of several chronic diseases [26,27]. The protective role is exerted through different pathways and the ability to inhibit glycative stress and dicarbonyl stress has been studied in different experimental models as reviewed [28].

## 2. Materials and Methods

### 2.1. Reagent 

Human colon epithelial cells CaCo-2 (ATCC^®^HTB-37™) were obtained from the American Type Culture Collection (Rockville, MD, USA). The compounds for cell cultures were purchased by Euroclone (Euroclone, Italy). Carboxy-H_2_DCFDA (C400) was supplied by Invitrogen (Invitrogen, Carlsbad, CA, USA). Goat polyclonal anti-AGE (AB9890) antibodies and rabbit polyclonal β-actin (A2066) were purchased by Sigma Aldrich (Sigma, St Louis, MO, USA). All other reagents have also been purchased from Sigma Aldrich (Sigma, St Louis, MO, USA).

### 2.2. Polyphenolic Extract 

In this study, Calville White Winter apples were used. These apples are included in the Regional Repertory of Agro-biodiversity of Marche (Italy) managed by Agency for Agrofood Sector Services of the Marche Region (ASSAM, Marche Region, Italy). For preparation of the extract, apples were sliced thinly, the pits were removed, and the pulp was lyophilized in a Heto Dry Winner 685 (Denmark) lyophilizer for 4 days. Freeze dried samples were pounded through a laboratory mill and then kept at −20 °C. Apples lyophilized (2 g) were treated with 80% methanol and 0.1% formic acid in agreement with previous studies [29,30]. The hydro-alcoholic extract has been used for HPLC separation and quantification of phenolic compounds and our results have been previously published [30] and showed in Table 1. 

To investigate the effect of apple polyphenols on cell-based assay, an aliquot of hydro-alcoholic extract has been used for solid-phase extraction (SPE) to remove polar non-phenolic compounds such as sugars and organic acids. CHROMABOND^®^ PA C18 cartridges (Carlo Erba Reagents s.r.l, Cornaredo, Italy) were used to extract phenolic compounds acid from the apple extract based on the method described by Saeidi et al. [31]. Briefly, the SPE cartridge was sequentially conditioned with 5 mL of n-hexane, 5 mL of methanol and 10 mL of double distilled deionized water without allowing the cartridge to dry. The filtrate was passed through the cartridge, washed by 8 mL water/methanol (90:10 *v/v*, adjusted at pH = 3 with concentrated HCl) to remove interferences and eluted with 4 mL HPLC grade methanol. Finally, the eluent (purified polyphenolic extract) was collected, concentrated using a rotary evaporator, dried, reconstituted with in sterile phosphate buffer saline (PBS). PBS-polyphenol extracts were stored under refrigeration (−20 °C) and used for cell incubation. Polyphenol yield of SPE has been evaluated as a balance between those initially found in hydro-alcoholic extract and those obtained in the retained and not retained fraction. Using Folin–Ciocalteu’s method [32], the results demonstrated that polyphenols in purified polyphenolic extract accounted for about 72% of those in the hydroalcoholic extract. 

### 2.3. Cell Model and Apple Polyphenols Treatment 

CaCo-2 cells were cultured at 37 °C in a humidified atmosphere containing 5% (*v/v*) CO_2_. The culture medium was constituted by Dulbecco’s minimal essential medium (DMEM) supplemented with 10 mM nonessential ammino acids, 10% (*v/v*) fetal bovine serum (FBS), 100 μg/mL streptomycin, 100 U/mL penicillin, 2 mM glutamine. Cellular vitality was measured using Trypan Blue 0.1% exclusion assay. Cells were treated with culture medium containing 25 mM (control cells) or 50 mM glucose (high glucose, HG) concentrations for 1 week [20], in the absence or in the presence of apple extract containing two concentrations of polyphenols (0.4 mmol GAE/L and 0.8mmolGAE/L) [30,33]. Medium was replaced two times a week.

### 2.4. Total Protein Quantification

The cells were detached from the plate with trypsin and centrifuged at 1200× *g* for 10 min. Pellets were rinsed twice with phosphate-buffered saline (PBS). The washed pellet was subsequently treated for 30 min on ice with a sodium-phosphate buffer pH 6.8 containing protease inhibitors (1.6 mM aprotinin, 0.08 mM bestatin, 2.08 mM 4-(2-aminoeethyl) benzenesulfonil fluoride hydrochloride, 0.3 mM pepstatin A, 0.03 mM E-64, 0.04 mM leupeptin) and 0.5% NP40 detergent, in order to obtain cell extracts. Cell lysates were subsequently centrifuged at 12,000× *g* for 15 min, at 4 °C. Supernatants were recovered and total protein concentration was measured by the Bradford assay. 

### 2.5. Advanced Glycation End Products (AGEs)

Fluorescent AGE levels were measured by reading the intrinsic fluorescence of AGE at 340 nm/420 nm (excitation and emission wavelengths respectively) on a microplate reader (Synergy microplate reader, BioTek Instruments, Inc.). The data are the result of the fluorescence intensity per mg of cell proteins [34]. 

For detection of total glycolaldehyde (GA)-modified proteins, Western blot analysis was performed. Cells extracts, containing 50 μg of protein, were run on 12.5% SDS-PAGE and then transferred to a polyvinylidene fluoride (PVDF) membrane. After blocking and washing, the membranes were incubated overnight with anti-AGE polyclonal goat (#AB9890 Merk Millipore, Burlington, MA, USA) at 4 °C. β-actin (A2066 Sigma-Aldrich, St. Louis, MO, USA) has been used as loading control. The membranes were then incubated with HRP-labeled secondary specific antibodies (A50-101P Dako, Santa Clara, CA, USA) for 1.30 h after being washed three times with Tris-buffered containing 0.1% Tween 20 (TBST). SuperSignal West Femto Maximum Sensitivity Substrate (Thermo Fisher Scientific, Waltham, MA, USA) was used for band development while the ChemiDoc XRS+System (Bio-Rad Laboratories, Hercules, CA, USA) were used to detect chemoluminescence. The chemiluminescent signal was analyzed by Image J software (Version 1.50i, National Institute of Health, Bethesda, MD, USA).

### 2.6. Intracellular ROS Levels

The intracellular ROS levels were evaluated using the probe carboxy-2,7-dichlorofluorescein diacetate (carboxy-H_2_DCFDA) (Invitrogen, Carlsbad, CA, USA) and detected by flow cytometry. The cells, resuspended at a final concentration of 0.5 × 10^6^ cells/mL, were incubated at 37 °C in preheated PBS containing 10 µM carboxy-H_2_DCFDA for 30 min in the dark. The cells were washed twice in PBS and then stained with 10 µg/mL propidium iodide (PI). The fluorescence of the cells was detected by cytofluorimetric analysis (Coulter EPICS XL, Beckman Coulter, CA USA), using an excitation wavelength of 488 nm. To assess ROS levels only on viable cells, cells that had absorbed PI and therefore had compromised cell permeability, were excluded from the analysis. The data were subsequently analyzed by the FCS Express Program (De Novo Software, CA, USA).

### 2.7. Lipid Peroxidation Products

The levels of malondialdehyde (MDA), the major lipid peroxidation products were evaluated as thiobarbituric acid reactive substances (TBARS) [35]. Briefly, one mL of 20% (*w/v*) trichloroacetic acid containing 0.8% (*w/v*) thiobarbituric acid (TBA) was added to each culture dish (60 mm diameter). The cells were scratched off and transferred to glass centrifuge tubes. After incubation at 95 °C for 45 min samples were cooled to room temperature in an ice bath for 10 min. All samples were centrifugated and the absorbance of the supernatant was evaluated on a microplate reader at OD 535 nm. The molar extinction coefficient of the (Malondialdehyde) MDA-TBA complex of 1.49 × 10^5^ M^−1^ cm^−1^ was used to calculate the amount of TBARS. Results are expressed as nmol MDA equivalents formed per mg protein.

### 2.8. Cell total Antioxidant Capacity

The total antioxidant capacity of CaCo-2 cells incubated in different experimental conditions was performed by Oxygen radical absorbance capacity (ORAC) assay. Briefly, Trolox standard and samples were added to wells in a black 96-well microplate. A volume of Fluorescein solution was added to each well (final concentration 0.08 mM) and incubated at 37 °C for 20 min before the addition of 17.5 mM freshly prepared 2,2′-Azobis(2-methylpropionamidine) dihydrochloride (AAPH) in working buffer. The decay of fluorescence at 530 nm was evaluated for 3 h in a microplate reader (BioTek Synergy HP, VT, USA) with excitation wavelength at 485 nm. The area under the fluorescence versus time curve was used for quantification [36]. Antioxidant capacity was expressed as mmol Trolox equivalents (TE)/10^6^ cells.

### 2.9. Quantitative Determination of Total Glutathione

The glutathione reductase (GR) recycling assay in the presence of 5,5-dithiobis (2-nitrobenzoic acid) (DTNB) was used to evaluate total levels of glutathione at 412 nm. A solution of GSH was used for the calibration curve [37]. Cells were trypsinized, washed twice in cold PBS, and centrifuged. Then the pellet was resuspended with 1% sulfosalicylic acid. All samples were incubated 30 min at 4 °C and after centrifugation at 2300× *g* for 2 min, the supernatant was recovered and analyzed for glutathione quantification. Finally, the pellet was resuspended with 1 M NaOH for recovery and used to quantify proteins using the Bradford method. Intracellular total glutathione is expressed as nmol/mg protein. 

### 2.10. Glyoxalase System Enzymatic Assay

Glyoxalase I activity was determined spectrophotometrically at 25 °C by monitoring the intermediate S-D-lactoylglutathione (SLG) formation at 240 nm for 1 min (ε = 2.86 mM^−1^ cm^−1^). The hemithioacetal is pre-formed in situ by incubation of 2mM GSH (freshly prepared) and 2 mM MGO in 100 mM phosphate-buffer pH 6.8. Samples were incubated at room temperature in the dark for 15 min. Then, 50μL of each sample were used. GlxI activity was expressed as µmol/min/mg protein [38]. 

To evaluate Glyoxalase II activity, the increase of GSH was studied for 1 min at 412 nm (ε = 13.6 mM^−1^ cm^−1^). Briefly, samples were incubated in the reaction mixture containing 100 mM MOPS buffer (pH 7.2), containing 0.8 mM SLG and 0.2 mM 5,5′- DTNB. GlxII activity was expressed as µmol/min/mg protein [39].

### 2.11. Statistical Analysis 

Data are expressed as the mean of measurements conducted separately ± Standard deviation. The Tukey-Kramer multiple comparison test or Kruskal-Wallis 1-way ANOVA were used to determine significant differences between treated and control cells.

## 3. Results

### 3.1. Effect of High Glucose and Polyphenols on Oxidative Stress

Table 1 summarizes polyphenol composition evaluated previously by HPLC analysis [30]; the hydroalcholicextract obtained from Calville White Winter apples was mainly composed of flavonols, in particular procyanidins, anthocyanins, and dihydrochalcones (phloridzin and phloretin-2-o-xyloglucoside) (Table 1). 

As shown in Table 2, we observed a significant increase in intracellular ROS and a significant decrease of total intracellular antioxidant capacity in cells incubated with HG compared with control cells. There was also an increase in MDA expressed as TBARS (*p* < 0.001). All these results support that our experimental conditions trigger glyco-oxidative stress of CaCo-2 cells.

In cells incubated in the presence of high glucose and apple polyphenol extract (0.4 and 0.8 mmol GAE/L) significant differences were observed. Lower levels of intracellular ROS and lipid peroxidation were observed, moreover, total antioxidant capacity was increased (Table 2). The protective effect was related to polyphenol concentration. These results demonstrate that polyphenol treatment protects CaCo-2 cells against oxidative stress. 

### 3.2. Effect of High Glucose and Polyphenols on AGEs Formation

**Figure 2:**
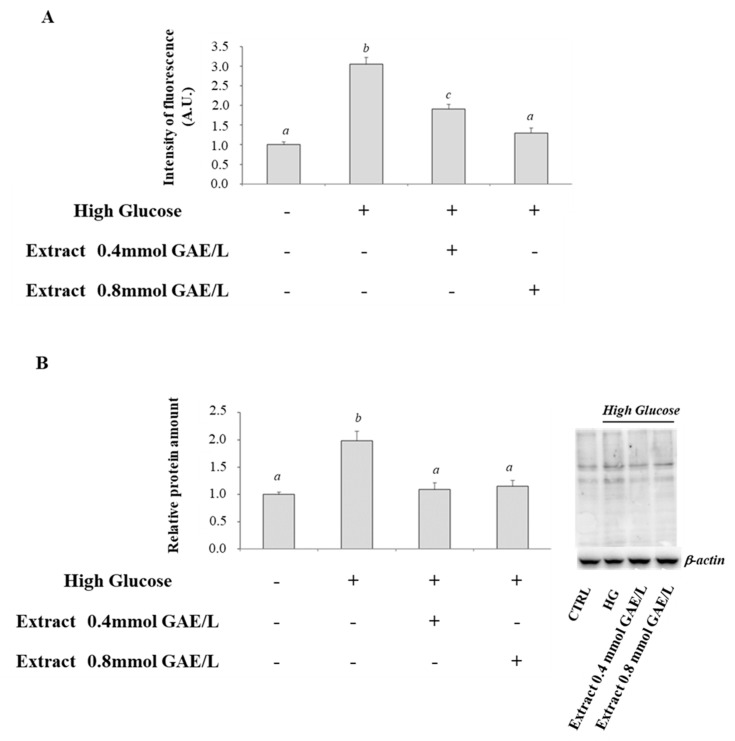
AGEs formation and GA-modified protein in HG-treated CaCo-2 cells in absence and in the presence of apple polyphenolic extract. (**A**) Levels of total fluorescent AGEs and (**B**) Representative western blot of GA-modified proteins and relative densitometric analysis, in control cells (25 mM glucose, Ctrl) or high glucose treated cells (50mM glucose) incubated in the absence (HG) or in the presence of apple polyphenolic extract (0.4 and 0.8 mmol GAE/L). Densitometric data are normalized on β-actin. Results are presented as mean ± SD of 5 determinations carried out in triplicate. Different letters a–c indicate significant statistic differences between samples (*p* < 0.05).

As shown in Figure 2, a significant increase in fluorescent AGEs formation and GA-modified proteins in cells incubated with HG compared with control cells was observed. HG plus polyphenolic extract treatment decreased significantly AGEs formation with respect to HG-treated cells. The effect on fluorescent AGEs was dependent on polyphenol concentration (Figure 2). 

### 3.3. Effect of High Glucose and Polyphenols on Glyoxalase System

**Figure 3:**
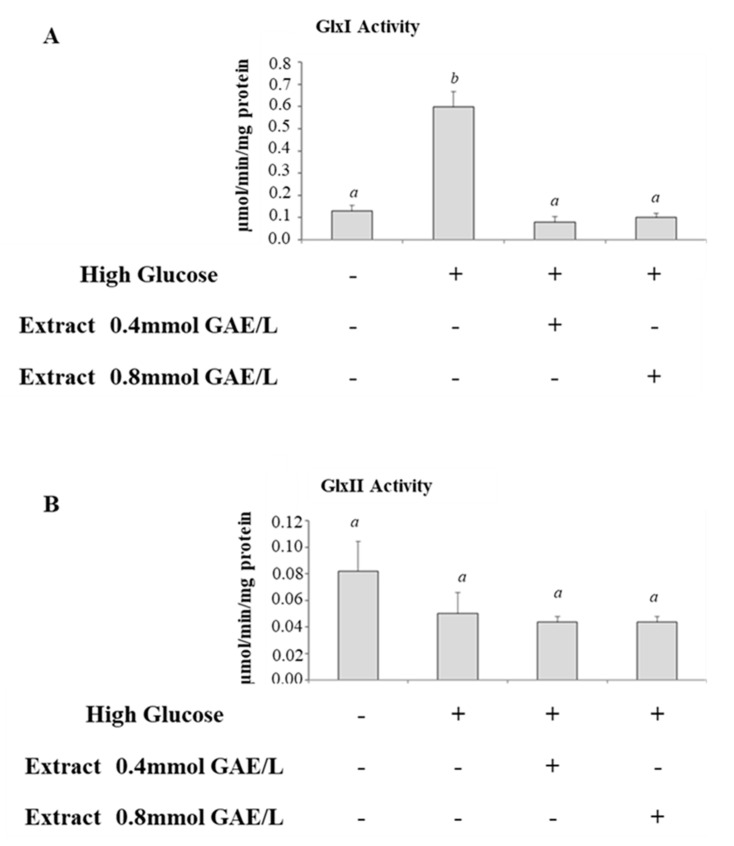
The glyoxalase system in HG-treated CaCo-2 cells incubated in the absence or in the presence of apple polyphenolic extract. (**A**) Glx I and (**B**) Glx II activity in intestinal CaCo-2 cells treated for one week with normal (25 mM) (Ctrl), or with high glucose (50 mM) concentrations in the absence (HG) or in the presence of polyphenolic extract (0.4 and 0.8 mmol GAE/L). Results are presented as mean ± SD of 5 determinations carried out in triplicate. Different letters a,b indicate significant statistic differences between samples (*p* < 0.05).

**Figure 4:**
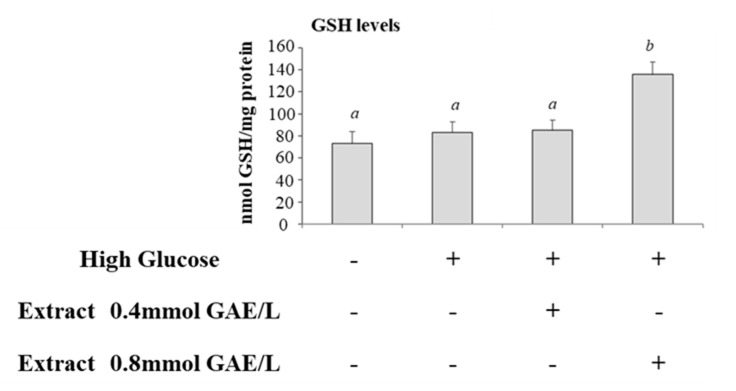
Glutathione level in HG-treated CaCo-2 cells in the absence or in the presence of apple polyphenolic extract Glutathione (GSH) level in control cells (25 mM glucose, Ctrl) or high glucose treated cells (50 mM glucose) incubated in the absence (HG) or in the presence of apple polyphenolic extract (0.4 and 0.8 mmol GAE/L). Results are represented as mean ± SD of 5 determinations carried out in triplicate. Different letters a,b indicate significant differences between samples (*p* < 0.05).

We studied the effects of incubation of cells with HG on glyoxalase system. Therefore, we compared the activities of GlxI and GlxII and levels of total glutathione in control cells and cells incubated with HG. GlxI activity increased significantly after HG treatment compared with control cells (Figure 3A), while GlxII activity was lower in HG-treated cells (Figure 3B). GSH levels were not significantly modified (Figure 4).

GlxI activity was lower in HG-treated cells incubated with polyphenolic extract and the activity was getting closer to control cells (Figure 3A); GlxII was not significantly modified after incubation with polyphenols (Figure 3B). Intracellular levels of GSH were increased in HG-treated cells incubated with polyphenols; the effect was significant at the higher concentration of polyphenols (Figure 4).

## 4. Discussion

Many “in vivo” and “in vitro” studies have shown that there is a strong correlation between a hyperglycemia condition and the formation of AGEs [40,41]. As previously demonstrated, intestinal cells are sensitive to glyco-oxidative stress when exposed to high glucose concentrations [20]. Glyco-oxidative stress was confirmed in this study with an increase of AGEs formation, increase of intracellular ROS production, lipid peroxidation, and a decrease of total antioxidant capacity. 

Glyoxalase system plays a key role against dycarbonyl stress as it maintains MGO at levels that are non-toxic to the cell. The glyoxalases system has always been studied as a pathway of MGO detoxification in a unique way, while in recent years some studies have considered the role of glyoxalases in the cellular redox signalling. The two enzymes involved (GlxI and GlxII) use GSH as a cofactor whose concentration must be considered as a factor closely related to the efficiency of the system itself. So, the increase in the MGO concentration and AGE, within the cell, may be due not only to increasing with high rates of glycolysis, but also to a decreased availability of GSH used under ROS overproduction condition. In light of this, the enzymatic activity of glyoxalases may be important in the cellular response to glyco-oxidative stress.

In our experimental model, under HG conditions, we observed a marked increase in GlxI activity coupled with a constant level of glutathione in comparison to the control. The high activity of the GlxI implies consumption of GSH which is however maintained at constant levels. Since GSH is a fundamental component in redox homeostasis, it is possible to assume that the cell must necessarily restore, as soon as possible, the amount consumed of GSH especially in conditions of chronic stress as in our experimental model. An increase in GlxI activity has been observed by other authors in HG treated cells and it has been suggested as a possible protection from high MGO formation in HG condition [42].

In our experimental conditions, despite the significant increase in GlxI activity, levels of AGEs were higher in HG treated cells. Other authors have demonstrated that the GlxI enzyme was ineffective to normalize MGO [43] in HG-treated cells. It has also to be stressed that AGEs formation does not only occur due to the increase of intracellular MGO but also as a consequence of glucose auto-oxidation and the non-enzymatic Maillard reaction and/or lipid peroxidation.

Contrasting results have been reported on the effects of incubation with high glucose levels on glyoxalase system in different experimental models. In vivo studies in streptozotocin induced diabetic rat, an increase in GlxI and GlxII activity in red blood cells and skeletal muscle was observed while the activity decreased in the liver [44,45]. Other authors have demonstrated that GlxI increased in glomeruli of diabetic mice, while decreased in renal cortex [46]. A decrease of GlxI activity has been shown in SH-SY5Y neuroblastoma cells, and on human brain microvascular endothelial cell line (IHEC) during hyperglycemia. Furthermore, the effects of high glucose on GLxI and GLxII differ. For instance, no effects on GlxII activity have been observed [47]. All these findings suggest that the effects of high glucose concentrations on glyoxalase enzymes are strongly tissue specific. 

Moreover, the activity of GlxII is about 10-fold lower than that of GlxI so it is the rate limiting enzyme of the MGO pathway [8]. GlxII is found in two isoforms, cytosolic and mitochondrial, which are differently regulated. The study by Scirè et al. [48] shows that acid phospholipids inhibit only the cytosolic form of GlxII and not the mitochondrial one, thus assuming a regulatory role of GlxII enzyme. It has also been shown that S-D-lactoylglutathione, when it accumulates in the cytoplasm, can enter into the mitochondria where it is used by mitochondrial GlxII [49]. Therefore, GlxII can represent a regulatory step of the glyoxalase pathway and for this reason the activity levels of GlxII do not always have the same trend as those of GLxI. Moreover, protein–protein interaction studies, performed using a validated in silico approach [50], have been shown that GlxII could catalyze protein S-glutathionylation using S-D-lactoylglutathione as substrate, on specific redox dependent proteins [39]. This capacity implies that S-D-lactoylglutathione formation from MGO and GlxII activity could play a relevant role in the regulation of cell metabolism and redox signaling.

In our experimental conditions, incubation with polyphenols during HG treatment was associated with a decrease of AGEs formation, ROS production, and lipid peroxidation evaluated as TBARS. Furthermore, an increase of total antioxidant capacity and GSH levels was observed. All these results demonstrate that apple polyphenols were able to counteract the glyco-oxidation induced by HG. Other studies have demonstrated the ability of fruit polyphenols to exert a protective effect against formation of AGEs [28,30,51,52]. There are many steps in ROS and AGEs production therefore different mechanisms may occur to delay or decrease intracellular ROS and AGEs formation [28,51]. Purified phenolic compounds can reduce glyco-oxidative stress through many pathways, including reduction of ROS production during the glycation process and trapping of dicarbonyl species [28,51]. A large amount of ROS is produced by the early stage of the Maillard reaction and Schiff bases produce ROS and RCS. Therefore, capturing ROS at the early stage of glycation can inhibit glycation. Moreover, polyphenolic compounds with specific chemical structural arrangement exert a high reactivity with MGO [53,54]. An MGO-trapping effect of some apple polyphenols (quercetin, phloretin and phloridzin) has been recently demonstrated [28,55]. 

In our study, the significant decrease of ROS formation and increase in GSH levels in cells treated with apple polyphenols during incubation with high glucose levels, was associated with a decrease of GlxI activity at values similar to those observed in control cells. In this picture it is possible to hypothesize a lower need to transform the MGO and this event can be correlated with the direct trapping activity of polyphenols on the MGO.

Other studies have reported conflicting results. Frandsen et al. [56] demonstrated that the flavonoids catechin, morin, and quercetin enhanced the glyoxalase pathway in neuronal cells in culture with an over-expression of GlxI, GlxII, increase of GSH and decrease the concentration of ROS. While other studies show that polyphenols such as curcumin, luteolin, myricetin, and kaempferol negatively modulate GlxI [57,58,59].

Different mechanisms could be advanced to explain the effect of apple polyphenols on GSH levels and glyoxalases in CaCo-2 cells exposed to high glucose levels. It has been suggested that modulating signaling pathways involved in cellular behavior, GSH synthesis, and expression of antioxidant enzymes could be involved as suggested by other authors [56]. In the glyoxalase pathway, GSH is involved, as cofactor, in the first reaction that converts MGO to D-lactate. In some cell models, an increase of intracellular concentration of GSH, modulates glyoxalase pathway. Apple contains several flavonoids as demonstrated in our study. Some authors have suggested that the increase in GSH levels in flavonoid treated cells could be related to transactivation of catalytical subunit promoter of the gamma-glutamyl-cysteine synthetase [60]. GSH is involved in cellular redox regulation as it can be used as a cofactor by enzymes involved in signal transduction [61,62], therefore our results suggest that polyphenol-mediated regulation of GSH levels could modulate cellular response in intestinal cells. Moreover, some flavonoids behave as natural inhibitors of GlxI [63]. 

We confirm that apple polyphenols exert a protective effect against oxidative stress and dicarbonyl stress in CaCo-2 cells. The effect was observed at concentrations ranging 4–8 × 10^−4^ mol/L. Polyphenols are the most abundant antioxidants in the diet [64]. It has been reported that bioavailability of polyphenols is related to structural properties of molecules. Total levels of polyphenolic compound are present in plasma at <1 µmol/L concentrations, but they are present in the stomach and intestinal lumen at much higher concentrations after consumption of foods and beverages rich in such compounds. For example, the dilution of 500 mg of polyphenols with the digestive bolus in the colon would give a local concentration of about 3 mM [65]. The poor intestinal absorption is responsible for luminal concentrations of phenolic compounds up to several hundred µmols in the gastrointestinal tract [66].

Previous studies have shown that incubation of intestinal cells with apple extracts results in a marked increase in intracellular concentration of some polyphenols such as catechin, caffeic acid, and epicatechin indicating that these compounds are able to penetrate cell membranes [33]. In addition, a number of phenolic compounds and their conjugates have been detected and identified in CaCo-2 cell lysates, confirming their active uptake and metabolism [67]. Further studies are necessary to investigate whether the effects of polyphenols on glyco-oxidative stress of CaCo2 cells are exerted by metabolites of apple polyphenols. In fact, the main metabolites of polyphenols have become the subject of manifold research studies and several bioactive roles have been demonstrated [68].

## 5. Conclusions

In conclusion in this work it has been highlighted that the MGO detoxification system is closely related to the redox balance of the cell even under conditions of glyco-oxidative stress. In particular, changes in the enzymes activity of the glyoxalase system may suggest an involvement of this metabolic pathway in the antioxidant cellular response.

The incubation with polyphenols during HG treatment is associated with the recovery of glyco-oxidative stress parameters demonstrating that apple polyphenolic extracts were able to counteract the glyco-oxidation, even with the involvement of the glyoxalase system. Therefore, the polyphenols contained in apples can have two beneficial properties for the cell. On the one hand they increase the antioxidant capacity and therefore predispose the cell to an advantageous condition to deal with chronic stress, on the other hand they work directly from antioxidants and trapping for harmful compounds such as MGO. Further studies are necessary to understand better the effects of high glucose and polyphenols on dicarbonyl stress and glyoxalase system in intestinal cells and the molecular mechanisms that could be involved.

## Figures and Tables

**Table 1:** Polyphenolic compounds in *Calville W.W*. apple. Data are expressed as mean value (mg/100 g fresh weight) (*n* = 5).

Class of Polyphenols	Polyphenolic Compound	mg/100g FW (Fresh Weight) Calville W.W.
	procyanidin B1	5.78
	procyanidin B2	229.68
	procyanidin trimer	88.74
Flavanols	procyanidin tetramer	11.87
	procyanidin pentamer	5.25
	±catechin	0.88
	epicatechin	2.37
Flavones	Luteolin-glycoside	0.01
Flavonols	Rutin + hyperin	3.13
	isoquercitrin	0.018
	reynoutrin	0.016
	guajaverin	0.011
	avicularin	0.027
Hydroxycinnamic acids	chlorogenic acid	0.53
Dihydrochalcones	phloretin-2-o-xyloglucoside	1.75
	phloridzin	5.55
Anthocyanins	cyanidin-3-o-galactoside	5.15
Total polypjenols		361 ± 11

**Table 2:** The oxidative stress in HG-treated CaCo-2 cells with or without polyphenols extract. Intracellular ROS production, total antioxidant capacity, lipid peroxidation, and total AGEs levels in control cells (25 mM glucose, Ctrl) or high glucose treated cells (50 mM glucose) incubated in the absence (HG) or in the presence of apple polyphenolic extract (0.4 and 0.8 mmol GAE/L). Results are represented as mean ± SD of 5 determinations carried out in triplicate. Different letters a–d indicate significant statistic differences between samples (*p* < 0.05).

	Intracellular ROS Production (Intensity of Fluorescence A.U.)	Antioxidant Capacity (µmol TE/10^6^ Cells)	Lipid Peroxidation (nmol/mg Protein)
CTRL	14 ± 2 ^a^	806 ± 161 ^a^	0.55 ± 0.21 ^a^
HG	29 ± 2 ^b^	286 ± 152 ^b^	3.01 ± 0.62 ^b^
HG + Extract 0.4 mmol GAE/L	15 ± 1 ^a^	592 ± 150 ^c^	0.92 ± 0.18 ^c^
HG+ Extract 0.8 mmol GAE/L	10 ± 1 ^c^	715 ± 157 ^d^	0.54 ± 0.08 ^a^
